# Supplementary material for: Contribution of Heparan Sulphate Binding in CCL21-Mediated Migration of Breast Cancer Cells
Source: Cancers (Basel). 2021 Jul 10;13(14):3462. doi: 10.3390/cancers13143462 (PMC8306094; doi:10.3390/cancers13143462)
Supplement: Supplementary file 1 [file cancers-13-03462-s001.zip › cancers-1230219-supplementary.pdf]

cancers

MDPI

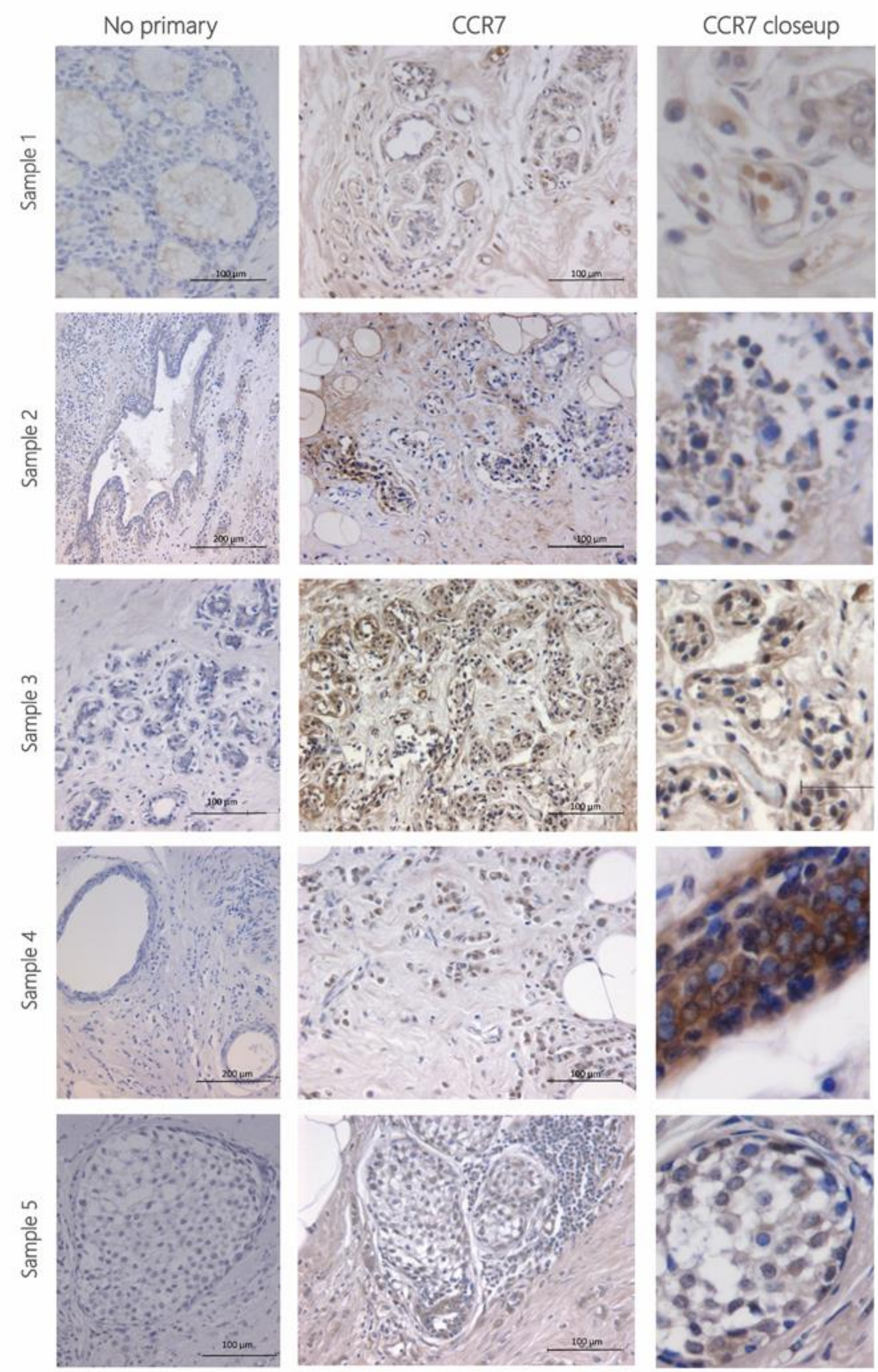

Supplementary figure 1: CCR7 staining in human breast cancer tissue.

4 µm sections from human breast cancer were stained for CCR7 (1:50) using the ImmPRESS polymer detection kit following no pre-treatment for antigen retrieval. Signal was developed using DAB (brown stain) and counterstained with haematoxylin (blue). Microscopy images were taken at 20x magnification. No primary antibody was used as a control, depicted in the left panel at 10x magnification. Patient details can be found in Table 1.

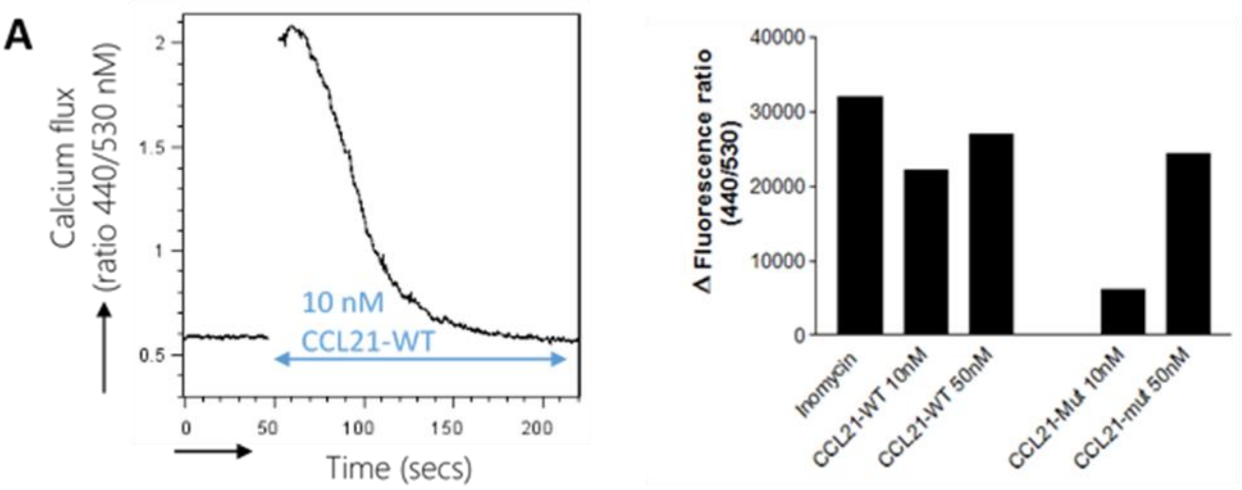

B

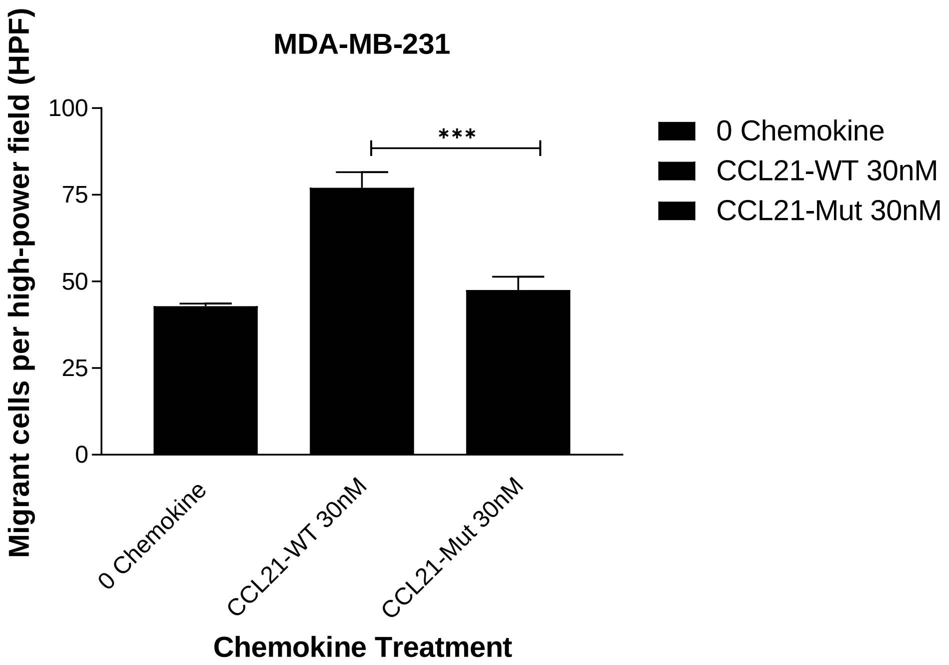

Supplementary figure 2: Biological activity of mutant and WT CCL21 in MDA-MB231 cells. (A) Calcium flux. MDA-MB-231 cells were loaded with Indo-1am for 30 minutes at 37°C before being run in the BD LSRFortessa™ X-20 cell analyser. Cells were then stimulated with different concentrations of wild type and mutated CCL21 and calcium flux was assessed. When the intracellular calcium is released, the Indo-1am binds it, changing its UV emission from 510 nm to 420 nm and thus increasing the ratio between these wavelengths (left). Fluorescence ratio was assessed, with ionomycin as positive control. **B) Trans-endothelial chemotaxis of MDA-MB231.** HMEC-1 cells were grown to confluence on the upper surface of 8µm pore filter, 72h prior to the assay. Chemokines were added to the lower well and

200,000 MDA-MB231 cells were added to the upper well and cells allowed to migrate for 16h at 37C. Migrated cells were quantified by counting average number of cells/HPF. N=3. ***, $p < 0.001$; **, $p < 0.01$; *, $p < 0.05$.

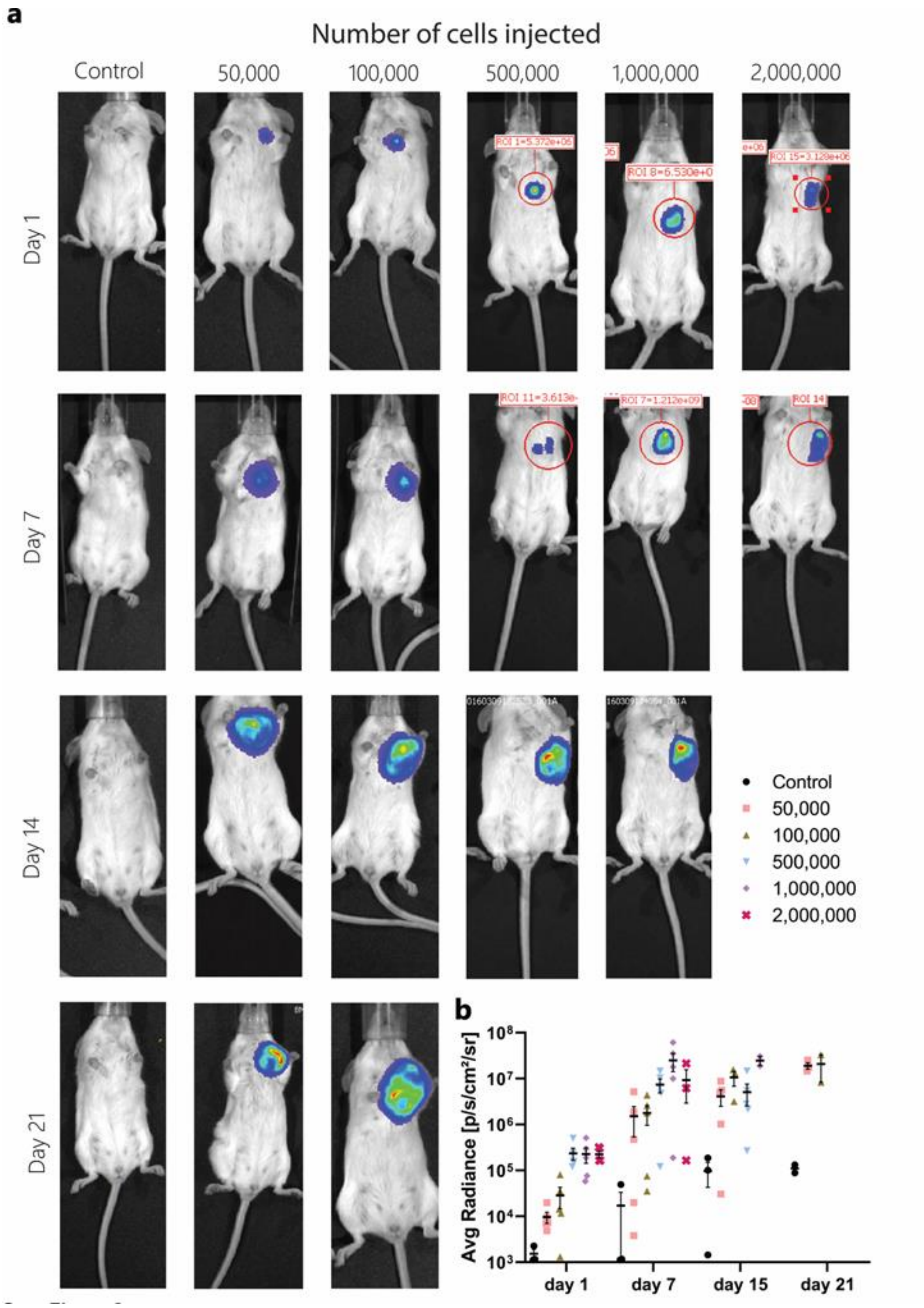

Supplementary Figure 3: Monitoring of tumour growth's progression during three weeks using IVIS spectrum. 5x10[4], 1x10[5], 5x10[5], 1x10[6] or 2x10[6] 4T1-Luc cells were injected into the mammary fat pad of five 8-week female BALB/c mice per group and tumour luminescence was imaged weekly for three weeks. (a) Representative images of each group are shown. (b) Quantification of the luminescence for each mouse.

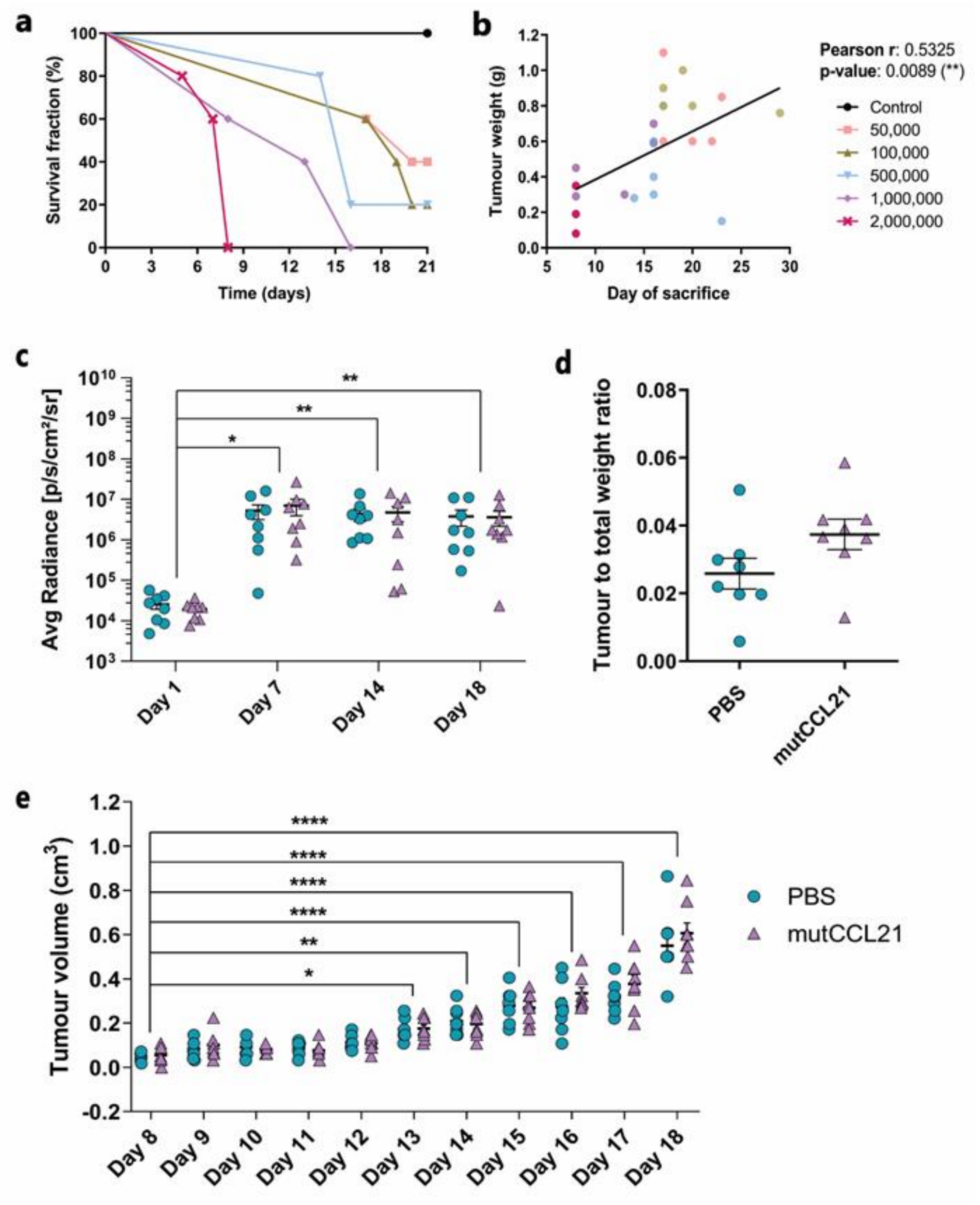

Supplementary figure 4: a) Kaplan-Meyer survival curve after injection with different 4T1-Luc cell numbers.

b) Correlation between the tumour weight and the day of sacrifice. Correlation plots display results from Spearman correlation tests and a linear regression line , with each injection group coloured separately (n=24)

c) Primary tumour luminescence was assessed weekly and on the day of sacrifice (d=18) using IVIS. Data represent the individual luminescence plus the mean ± SEM of 8 mice per group; and statistical significance was calculated using a Kruskal–Wallis test with Dunn's post hoc test.

d) Excised primary tumour weight on day of sacrifice was normalised by the mouse total weight. Data represent the individual mouse weight ratio plus the mean ± SEM of 8 mice per group; and statistical significance was calculated using a Mann-Whitney test.

e) Primary tumour dimensions were recorded daily using calipers. Data represent the individual tumour volume plus the mean ± SEM of 8 mice per group; and statistical significance was calculated using a Kruskal–Wallis test with Dunn's post hoc test.
